# Web renewal counting processes and their applications in insurance

**DOI:** 10.1186/s13660-018-1854-0

**Published:** 2018-09-25

**Authors:** Rong Li, Xiuchun Bi, Shuguang Zhang

**Affiliations:** Department of Statistics and Finance, University of Science and Technology of China, Hefei, China

**Keywords:** 62P05, 62E05, 60K30, 60K05, Renewal processes, Dependence, Web Markov skeleton processes, Precise large deviations, Moderate deviations

## Abstract

This paper investigates a nonstandard renewal counting process with dependent inter-arrival times-web renewal process. Several limit properties, including the tail of the exponential moment which is a crucial condition in many situations, are obtained. Then the results are applied in insurance to derive precise large deviations and moderate deviation formulas for the aggregate amount of claims.

## Introduction

Renewal processes are important counting processes and are used in various fields. In this paper, we investigate a nonstandard renewal counting process with nonindependent inter-arrival times $T_{1},T_{2},\dots$. The motivation of this paper comes from web Markov skeleton processes (WMSPs for short).

Intuitively, a WMSP is a jump process and also a Markov skeleton process such that, for the given information of its skeleton, the time slots between jumps are conditionally independent of each other. The dynamics of a WMSP can be described as follows:

1.1 $$ X_{0} \stackrel{\tilde{T}_{0} }{ \longrightarrow} X_{1}\stackrel{\tilde {T}_{1} }{ \longrightarrow}\cdots X_{n} \stackrel{\tilde{T}_{n} }{\longrightarrow}\cdots, $$

where $\{X_{n}, n \geq0\}$ is a Markov chain with state space E, and $\{ \tilde{T}_{n}, n \geq0\}$ is the set of time slots between adjacent jumps (see Liu et al. [21] and Ma et al. [22] for details).

WMSPs were found very useful in various natural and social sciences, such as finance, queueing theory, insurance and other related fields. For instance, we consider its applications in insurance. Let E be a collection of insurance policies, $X = \{X_{n}, n \geq0\}$ describe the transition behaviors of claims between policies, which forms a Markov chain with state space E, $\{Y_{n}, n \geq1\}$ and $T= \{T_{n}, n \geq1\}$ represent claims sequence and inter-arrive times of claims, respectively. The inter-arrive time between two claims is a random variable which may depend on the information of the current policy or claim and some other related policies or claims. For example, in the automobile insurance and the property insurance, the previous year’s claim times and claim sizes are important factors which influence the insurance purchasing, especially the premium for the next year, in other words, the distribution of the waiting time for the next large claim depends on the times and sizes of recent claims (claims during the current year for instance). Let $\sigma(Y)=\sigma (Y_{n}, n \geq1)$ be the sigma algebra generated by $\{Y_{n}, n \geq1\}$, then the following structure:

1.2 $$ \Pr\bigl(T_{n} > t|\sigma(Y)\bigr)= \Pr(T > t|Y_{n-k} > x,\dots,Y_{n} > x),\quad 1\leq k < n, $$

is a practical dependent structure, and the aggregate amount of claims,

1.3 $$ S_{t}=\sum^{N_{t}}_{i=1}Y_{i},\quad t\geq0, $$

forms a nonstandard renewal risk process, we called a web renewal risk process, which is an inevitable factor when measuring the risk, pricing the premium or other related behaviors for an insurance company, where

1.4 $$ N_{t}=\sup_{n}\{n\geq1: T_{1}+\cdots+T_{n}\leq t\},$$

is the renewal counting process, we called a web renewal process.

Suppose that $\lambda(t)=\mathbf {E}N_{t}\rightarrow\infty$ as $t\rightarrow \infty$. If the inter-arrival times $T_{1},T_{2},\dots$ form a sequence of independent identically distributed (i.i.d.) random variables, then (1.4) is the standard renewal process which is an important counting process in many applications, such as renewal risk model in risk theory. Some important limit properties of the standard renewal process, such as convergence and the limit distribution, have been extensively investigated in much literature (see Ross [24], Kaas and Tang [10], Ng et al. [23], Tang and Tsitsiashvili [28], among many others). But there are few results for the corresponding web renewal process. These are what we are going to study.

The rest of the paper is organized as follows. Section 2 gives the main results for $N_{t}$ and the proofs after some preliminaries. Section 3 presents some applications in insurance and derives the results of precise large deviations and moderate deviations for the web renewal risk process $S_{t}$. Section 4 concludes this paper. Some proofs are provided in the appendix.

## Main results and discussions

In this section we will give several limit properties of the counting process $N_{t}$.

A sequence $T_{n}, n \geq1$ of random variables is M-dependent, where M is a positive integer, if $T_{1},T_{2}, \dots, T_{i}$ is independent of $T_{j},T_{j+1}, \dots$ for $|j-i|>M$. Now we are in a position to state our main results.

### Theorem 2.1

*For the web renewal counting process* $N_{t}, t\geq0 $, *if* $T_{n}, n \geq 1$ *is a k*-*dependent sequence of identically distributed nonnegative random variables with common mean* $1/\lambda\in(0,\infty)$ *and finite variance*. *Then*

2.1 $$ \frac{N_{t}}{t}\rightarrow\lambda\quad\textit{a.s.} $$

*and*

2.2 $$ \frac{\mathbf {E}[N_{t}]}{t}\rightarrow\lambda. $$

In fact, it is not difficult to derive (2.1) and (2.2) from Theorems 4 and 7 in Korchevsky and Petrov [13], and Ross [24]. We omit it here.

### Remark 2.1

The constraint on $T_{n}, n \geq1$, M-dependent, is for tractability, and is also natural. Consider the dependent structure (1.2), if $Y_{n}, n \geq1$, is an i.i.d. or weakly dependent sequence, then it is easy to ensure that $T_{n}, n \geq1$, is a sequence of k-dependence under some conditions.

The following condition is crucial for a counting process in most applications.

### Assumption 2.1

For every real number $a > 1/\mathbf {E}[T]$, there exists a constant $b > 1$, such that

2.3 $$ \lim_{t\rightarrow\infty} \sum_{n>at}b^{n} \Pr (N_{t}\geq n )=0. $$

Kočetova et al. [12] proved that Assumption 2.1 is satisfied for the standard renewal counting process.

### Proposition 2.1

(Kočetova et al. [12])

*Let the renewal counting process* $N_{t}$ *be defined in* (1.4) *with a sequence* $T,T_{1},T_{2},\dots$ *of i*.*i*.*d*. *nonnegative r*.*v*.*s*. *Then*:

(i) *if* $\mathbf {E}T=1/\lambda<\infty$, *then* $N_{t}$ *satisfies Assumption *2.1;
(ii) *if* $\mathbf {E}T=\infty$, *then*, *for every* $a>0$, *there exists* $b>1$ *such that Eq*. (2.3) *holds*.

They also considered the applications of their result in insurance mathematics.

In the case of the standard renewal counting process with the finite mean $\mathbf {E}T=1/\lambda<\infty$, Assumption 2.1 is equivalent to the following assumption.

### Assumption 2.2

(Leipus and Šiaulys [14])

For every $\delta>0$, there exists $\epsilon> 0$ such that

$$ \lim_{t\rightarrow\infty} \sum_{n>(1+\delta)\lambda t}(1+\epsilon )^{n}\Pr (N_{t}=n )=0. $$

Assumption 2.2 is one of the crucial requirements for the counting process $N_{t}$ in the paper of Leipus and Šiaulys [14]. Furthermore, in this case, Assumption 2.2 implies the following assumption mentioned by Klüppelberg and Mikosch [11].

### Assumption 2.3

(Klüppelberg and Mikosch [11])

There exist positive *ϵ* and *δ* such that

$$ \mathbf {E}\bigl((1+\epsilon)^{N_{t}}1_{\{N_{t}> (1+\delta)\lambda(t)\}} \bigr)= \sum _{n>(1+\delta)\lambda(t)}(1+\epsilon)^{n}\Pr (N_{t}=n ) \rightarrow0, $$

as $t\rightarrow\infty$.

Assumption 2.3 is an essential condition in their paper.

The importance of the above-mentioned statements can also be found in Kaas and Tang [10], Ng et al. [23], Tang and Tsitsiashvili [28], Wang and Wang [29], Shen et al. [25] and others therein.

Fu and Shen [6] proved the following key lemma when they considered moderate deviations for sums of claims in a size-dependent renewal risk model.

### Lemma 2.1

(Fu and Shen [6])

*Let* $N_{t}$ *be the standard renewal process*, $\tau_{n}=\sum^{n}_{i=1}T_{i}, n\geq1$ *be the arrival times*. *Then*, *for any* $\delta>0$ *and some* $c > 0$

2.4 $$ \lim_{t\rightarrow\infty} \sum_{n>\lambda t+\delta b(t)}c^{n} \Pr (\tau_{n}\leq t )=0, $$

*where* $1/\lambda$ *is the common mean of inter*-*arrival times and* $b(t)$ *is a positive function satisfying* $b(t)/t\rightarrow0$ *as* $t\rightarrow\infty$.

It can be seen that the property of Assumption 2.1 is very important. We will prove that Eqs. (2.3) and (2.4) also hold for the web renewal counting process.

### Theorem 2.2

*Let* $T_{n}, n \geq1$, *be a sequence of identically distributed nonnegative random variables with common mean* $\mathbf {E}T\in(0,\infty]$ *such that* $T_{n}, n \geq1$ *is k*-*dependent for any positive integer* $k\geq1$. *Then*

(i) *if* $\mathbf {E}T=1/\lambda<\infty$, *then*
  1. 2.5 $$ \lim_{t\rightarrow\infty} \sum_{n>at}b^{n} \Pr (N_{t}\geq n )=0 $$
    *holds for every* $a > (k+1)\lambda$ *and some* $b > 1$;
  2. 2.6 $$ \lim_{t\rightarrow\infty} \sum_{n>\lambda t+\delta b(t)}c^{n} \Pr (N_{t}\geq n )=0 $$
    *holds for any* $\delta>0$, *some* $c>0$ *and a positive function* $b(t)$ *satisfying* $b(t)/t\rightarrow0$ *as* $t\rightarrow\infty$.
(ii) *if* $\mathbf {E}T =\infty$, *then*
  1. (2.5) *holds for every* $a > 0$ *and some* $b > 1$;
  2. (2.6) *holds under the same condition*.

Theorem 2.2 is an extension to the Lemma 3.3 in Bi and Zhang [2], which is a key lemma in the proof of their main results.

### Proof

We next give the proof of Theorem 2.2.

(i) For $\mathbf {E}T=1/\lambda<\infty$, we have $\Pr(T\leq t)\leq e^{yt}\mathbf {E}[e^{-yT}]$ for any $t>0, y >0$. Let $m(y)=\mathbf {E}e^{-yT}\in(0,1)$, we derive

$$\begin{aligned} \varphi_{a,b}(t):&= \sum_{n>at}b^{n} \Pr (\tau_{n}\leq t ) \leq\sum_{n>at}b^{n} \Pr \biggl(\sum_{1\leq1+(k+1)i\leq n}T_{1+(k+1)i}\leq t \biggr) \\ &\leq e^{yt}\sum_{n>at}b^{n} \bigl(m(y)\bigr)^{\frac{n-1}{k+1}}=\frac {e^{yt}}{(m(y))^{1/(k+1)}}\sum _{n>at}\bigl(b^{k+1}m(y)\bigr)^{\frac{n}{k+1}} \end{aligned}$$

for all real $a> (k+1)\lambda, b>1$, and $t>0$. $b^{k+1}m(y)<1$ for $b^{k+1}\in[1,\frac{1}{10}(1+\frac{9}{m(y)})]$, hence we find that, for such *b*

2.7 $$ \varphi_{a,b}(t)\leq \frac{1}{(m(y))^{1/(k+1)}}\frac{1}{1-b^{k+1}m(y)}\exp \biggl\{ yt \biggl(1+\frac{a\ln b^{k+1}}{(k+1)y}+ \frac{a\ln m(y)}{(k+1)y} \biggr) \biggr\} . $$

Taking the inequality $|e^{-v}-1|\leq v, v>0$, and the Lebesgue dominated convergence theorem into consideration, we get

2.8 $$\begin{aligned} \lim_{y\to0+}\frac{\ln m(y)}{y} &= \lim _{y\to0+}\frac{\ln(1+m(y)-1)}{y}=\lim_{y\to0+} \frac{ (m(y)-1)}{y} \\ &= \int_{0}^{\infty}\lim_{y\to0+} \frac{e^{-yu}-1}{y} \,d\Pr(T\leq u)=-\mathbf {E}T=-\frac{1}{\lambda}. \end{aligned}$$

Hence, there exists $y^{*}>0$, for which

$$a\frac{\ln m(y^{*})}{y^{*}}\leq-\frac{(k+1)}{2}-\frac{a}{2\lambda}. $$

For such $y^{*}$, $t>0$, and $b^{k+1}\in(1,\frac{1}{10}(1+\frac {9}{m(y^{*})})]$, we get

$$\varphi_{a,b}(t)\leq\frac{1}{(m(y^{*}))^{1/(k+1)}}\frac {1}{1-b^{k+1}m(y^{*})}\exp \biggl\{ y^{*}t \biggl(\frac{a\ln b^{k+1}}{(k+1)y^{*}}-\frac{a-(k+1)\lambda}{2(k+1)\lambda} \biggr) \biggr\} . $$

There also exists $\hat{b}=\hat{b}(a,y^{*})$ such that

$$\frac{a\ln\hat{b}^{k+1}}{y^{*}}< \frac{a-(k+1)\lambda}{4(k+1)\lambda}. $$

Hence, for some special positive constant $y^{*}$ and $t>0$, we have

$$\varphi_{a,\hat{b}}(t)\leq\frac{1}{(m(y^{*}))^{1/(k+1)}}\frac {10}{1-m(y^{*})}\exp \biggl\{ - \frac{a-(k+1)\lambda}{4(k+1)\lambda}y^{*}t \biggr\} \to0,\quad t\to\infty. $$

We obtain (2.5). Equation (2.6) can be proved similarly.

(ii) Let $\mathbf {E}T=\infty$. From estimate (2.7) it follows, for all positive $t, a$ and all $b^{k+1}\in[1,\frac{1}{10}(1+\frac {9}{m(y)})]$, that

2.9 $$ \varphi_{a,b}(t)\leq\frac{1}{(m(y))^{1/(k+1)}}\frac {10}{1-b^{k+1}m(y)} \exp \biggl\{ yt \biggl(1+\frac{a\ln b^{k+1}}{(k+1)y}+\frac{a\ln m(y)}{(k+1)y} \biggr) \biggr\} . $$

The r.v. $\min{\{T, r\}}$ has finite mean for every real positive number *r*. According to (2.8), we can get

$$\mathop{\overline{\lim}}_{y\to0+}\frac{\ln m(y)}{y}\leq\mathop{\overline{\lim }}_{y\to0+}\frac{\ln \mathbf {E}e^{-y\min{\{T, r\}}}}{y}=-\mathbf {E}\min{\{T, r\}}. $$

Hence

$$\lim_{y\to0+}\frac{\ln m(y)}{y}=-\infty, $$

and there exists a positive $y^{*}=y^{*}(a)$ such that, for $0< y\leq y^{*}$, we have

$$a\ln m(y)\leq-3 (k+1)y. $$

Estimate (2.9) implies that

2.10 $$ \varphi_{a,b}(t)\leq\frac{1}{(m(y))^{1/(k+1)}}\frac {10}{1-b^{k+1}m(y)} \exp \biggl\{ y^{*}t \biggl(\frac{a\ln b^{k+1}}{(k+1)y^{*}}-2 \biggr) \biggr\} $$

for $a>0, 1< b\leq\frac{1}{10}(1+\frac{9}{m(y^{*})}), t>0$, and some positive constant $y^{*}=y^{*}(a)$. Now after a suitable choice of $b=\hat {b}=\hat{b}(a,y^{*})$, the first statement follows from the last estimate. The second statement can be obtained through the similar method. □

### Remark 2.2

In fact, the above proof implies that Theorem 1(i) in Kočetova et al. [12] and Lemma 3.3 in Bi and Zhang [2] are direct corollaries of Theorem 2.2.

## Applications in insurance

In this section, we consider some applications of the main results in insurance. We investigate the precise large deviations and moderate deviations formulas for the web renewal risk process (1.3), where the claims $\{Y_{n}, n\geq1\}$ are identically distributed and nonnegative random variables (r.v.s) with the common distribution function (d.f.) $F(x)=\Pr(Y\leq x)$ and the finite mean $\mathbf {E}Y=\mu$, and the inter-arrival times $T_{n}, n\geq1$ depended on $\{Y_{n}, n\geq1\}$ through dependent structure (1.2)

We restrict our interest to the case of heavy-tailed distribution. A nonnegative $r.v$. *Y* or its distribution function $F(x)$ on $[0,\infty)$ is said to be heavy-tailed, if its moment generating function satisfies $\mathbf{E}[e^{sY}]=\infty$ for all $s > 0$. One of the useful classes of heavy-tailed distributions is the class $\mathcal{C}$ of consistent variation distributions. By definition of class $\mathcal{C}$, a distribution F on $[0,\infty )$ is said to be a consistent variation if $\overline{F}(x) > 0$, for all $x \geq0$, and the relation

$$\lim_{y\uparrow1}\limsup_{x\rightarrow\infty}\frac{\overline {F}(xy)}{\bar{F}(x)}=1 $$

holds.

Following Tang and Tsitsiashvili [28], we recall the useful functional index $J^{+}_{F}$, which is the upper Matuszewska index of *F*. Let *F* be a distribution function on $(-\infty,+\infty)$ and set $f = 1/\overline{F}$. Let $J^{+}_{F}$ be the infimum of those *J* for which there exists a constant $C = C(J)$ such that, for each $y_{0}>1$, the relation

$$\frac{f(xy)}{f(x)}\leq C\bigl(1+o(1)\bigr)y^{J} $$

holds uniformly in $y \in[1,y_{0}]$. The quantity $J^{+}_{F}$ defines the upper Matuszewska index of the function *f*, and by Theorem 2.1.5 and Corollary 2.1.6 in Bingham et al. [3], it coincides with

$$J^{+}_{F}=\inf\biggl\{ -\frac{\log\overline{F}_{*}(y)}{\log y}: y>1\biggr\} \quad \mbox{with } \overline{F}_{*}(y)=\liminf_{x\rightarrow\infty}\frac{\overline {F}(xy)}{\overline{F}(x)}. $$

From Proposition 2.2.1 in Bingham et al. [3], we see that, for every $p >J^{+}_{F}$, there are two positive constants *C* and $x_{0}$ such that

3.1 $$ \frac{\overline{F}(x)}{\overline{F}(xy)}\leq C y^{p}, \quad\mbox{for } xy \geq x \geq x_{0}. $$

Then one can easily derive the relation

3.2 $$ x^{-p}=o\bigl(\overline{F}(x)\bigr),\quad x\rightarrow\infty, $$

holds for all $p >J^{+}_{F}$. See also Lemma 3.5 in Tang and Tsitsiashvili [28].

For convenience, we introduce the following notations throughout this paper.

- For two positive functions $f(x)$ and $g(x)$, we write
  $$\begin{aligned} &f(x)\sim g(x)\quad \mbox{if } \lim_{x\rightarrow\infty}\frac{f(x)}{g(x)}=1, \\ &f(x)\lesssim g(x)\quad \mbox{if } \limsup_{x\rightarrow\infty}\frac{f(x)}{g(x)} \leq1. \end{aligned}$$
- For two positive bivariate functions $f(\cdot,\cdot)$ and $g(\cdot,\cdot)$, we say that $f(x,t)\lesssim g(x,t)$, as $t\rightarrow\infty$, holds uniformly in $x\in\Delta _{t}\neq\emptyset$, if
  $$\limsup_{t\rightarrow\infty}\sup_{x\in\Delta_{t}}\frac {f(x,t)}{g(x,t)} \leq1. $$
- For a distribution function $F(x)$ with finite mean $\mu>0$, set $\overline{F}(x)\equiv1-F(x)$ as the corresponding survival function of it.

### Precise large deviations

The precise large deviation of random sums has been extensively investigated in much literature since it was initiated by Klüppelberg and Mikosch [11], for example, Kaas and Tang [10], Tang [27], Lin [17], Baltrūnas et al. [1], Chen and Zhang [5], Liu [18], Li et al. [15], Chen and Yuen [4], Bi and Zhang [2], Shen et al. [25], Yang and Sha [30], Guo et al. [8], Hua et al. [9] and Liu et al. [20], among many others.

In order to formulate the precise large deviations results that, for any given $\delta> 0$,

3.3 $$ \Pr(S_{t}-\mu\lambda t>x)\sim\lambda t \overline{F}(x),\quad t\rightarrow\infty, $$

holds uniformly for all $x\geq\delta t$, $i.e$.,

$$ \limsup_{t\rightarrow\infty x\geq\delta t} \biggl\vert \frac{\Pr (S_{t}-\mu\lambda t>x)}{\lambda t \overline{F}(x)}-1 \biggr\vert =0, $$

one needs some constraint conditions on the dependent structure for our model.

#### Assumption 3.1

For $n \geq1$, $T_{n}$ was dependent on $Y_{n-k},\dots,Y_{n-1}, Y_{n}$, and independent of $Y_{1}, Y_{2}, \dots, Y_{n-k-1}, Y_{n+1},\dots$ such that the sequence $T_{n},n\geq1$ is *k*-dependent, $k\geq1$. There is an r.v. $T^{*}\ge0$ s.t. $T_{n}$ conditional on $(Y_{n-k} > x,\dots,Y_{n} > x)$ is stochastically bounded by $T^{*}$ for all $x > 0$ large enough, i.e. there is a constant $x_{0} > 0$ such that

3.4 $$ \Pr(T_{n} > t|Y_{n-k} > x, \dots,Y_{n} > x) \leq\Pr\bigl( T^{*} > t\bigr),\quad 1\leq k< n, $$

for all $x > x_{0}$ and $t\geq0$.

Then we have the following result for the web renewal risk process.

#### Theorem 3.1

*Consider the web renewal risk process* (1.3) *with i*.*i*.*d*. *claims*. *In addition to Assumption* 3.1, *suppose that* $F\in \mathcal{C}, \mathbf {E}[T] =1/\lambda\in(0,\infty)$ *and* $\operatorname {\mathbf {Var}}[T]<\infty$. *Then*, *for any given* $\delta> 0$, (3.3) *holds uniformly for all* $x\geq\delta t$.

If we select $k=1$ in Assumption 3.1, then we get the result of Theorem 2.1 in Bi and Zhang [2]. We restate it as a corollary of Theorem 3.1.

#### Corollary 3.1

(Bi and Zhang [2])

*Consider the aggregate amount of claims* (1.3) *with i*.*i*.*d*. *claims sequence*. *In addition to Assumption* 3.2, *suppose that* $F\in \mathcal{C}, \mathbf {E}[T] =1/\lambda\in(0,\infty)$ *and* $\operatorname {\mathbf {Var}}[T]<\infty$. *Then*, *for any given* $\delta> 0$, (3.3) *holds uniformly for all* $x\geq\delta t$.

#### Assumption 3.2

(Bi and Zhang [2])

(3.4) holds when $k=1$ in Assumption 3.1.

The proof of Theorem 3.1 is similar to that of Corollary 3.1. We omit it here. Pay attention that Theorem 2.2 is one of the key conditions for the proof and Theorem 3.1 really extends Corollary 3.1.

#### Remark 3.1

For the mutually independent claims $\{Y_{n}, n\geq1\}$, it is easy to construct an example that $T_{n},n\geq1$ is *k*-dependent under the Assumption 3.1. But if $\{Y_{n}, n\geq1\}$ are not mutually independent, it is hard to construct such sequence of *k*-dependence only under the Assumption 3.1.

Note that extended negatively dependence is a kind of weakly dependent structure and covers a wide range of dependence structures (see Liu [19] for more details). A sequence of random variables $\{X_{k}; k\in\mathbb{N}\}$ is said to be extended negatively dependent (END) if for each *n* and all $x_{1},\ldots,x_{n}$, there exists a constant $M>0$, independent of *n*, such that

3.5 $$ P(X_{1}\leq x_{1},\ldots,X_{n}\leq x_{n})\leq M\prod^{n}_{k=1}P(X_{k} \leq x_{k}) $$

and

3.6 $$ P(X_{1}>x_{1},\ldots,X_{n} > x_{n})\leq M\prod^{n}_{k=1}P(X_{k} > x_{k}). $$

If both (3.5) and (3.6) hold with $M = 1$, then the random variables are negatively dependent (ND); if both (3.5) and (3.6) hold in the reverse direction with $M = 1$, then the random variables are positively dependent (PD).

If the risks form an extended negative dependent (END) sequence, we need additional conditions to obtain the results of precise large deviations, which will be considered in the next subsection.

### Moderate deviations

Moderate deviations extend precise large deviations through extend *x*-region. Note that the *x*-region in Theorem 3.1 is taken as $[\delta t,\infty)$. It is natural to ask whether (3.3) can still hold for $x\in[\gamma b(t), \infty)$ with $b(t)/t\rightarrow0$ as $t\rightarrow\infty$, and if it can, what conditions are appropriate. Similar problems were partly studied by Shen and Zhang [26] for a risk model based on the customer-arrival process, by Gao [7] and Liu [19] for the standard renewal risk model with independent and dependent claims, respectively and by Fu and Shen [6] for the sums of consistently varying tailed claims in a size-dependent renewal risk model.

Taking Theorem 2.2 into consideration, we obtain the following result for the web renewal risk process proposed above.

#### Theorem 3.2

*Consider the web renewal risk process* (1.3) *with END claims*. *In addition to Assumption* 3.2 *with* $\mathbf {E}T^{*} <\infty$, *suppose that* $F\in\mathcal{C}$, $\mathbf {E}T=1/\lambda<\infty$, $\mathbf {E}|Y|^{\beta}<\infty$ *for some* $\beta>\alpha>1$ *and* $\operatorname {\mathbf {Var}}[T]<\infty $. *If*

3.7 $$ \frac{N_{t}-\lambda t}{b(t)}\stackrel{P}{\longrightarrow} 0,\quad t\rightarrow \infty, $$

*then*, *for any given* $\gamma>0$, *the result*

3.8 $$ \Pr(S_{t}-\mu\lambda t>x)\sim\lambda t \overline{F}(x),\quad t\rightarrow \infty, $$

*holds uniformly for all* $x\geq\gamma b(t)$, *where* $b(t)=a(\lambda t)$, *and a*(*t*) *is a positive function satisfying the conditions in Remark *3.2.

#### Proof

See the Appendix. □

#### Remark 3.2

Throughout, we suppose that $a(t),t\geq0$ is a positive function satisfying the following conditions:

- $a(t)< C t$ for *t* large enough and a positive constant *C*;
- $\lim_{t\rightarrow\infty}\frac{a(t)}{a([t])}=1$;
- $\lim_{t\rightarrow\infty}\frac{n(\log n)^{\alpha }}{a(n)^{\alpha}}=0, 1<\alpha< \min\{2, J^{+}_{F}\}$,

where $J^{+}_{F}$ is the upper Matuszewska index of a distribution *F* on $[0,\infty)$ (Tang and Tsitsiashvili [28] and Bingham et al. [3]).

Taking $a(n)=n^{1/\alpha}(\log n)^{2}$, then $a(n)$ satisfies the conditions in Remark 3.2 and $b(t)/t\rightarrow0$ as $t\rightarrow\infty$.

Theorem 3.2 is an extension to Theorem 2.1 in Fu and Shen [6]. Furthermore, we have

#### Theorem 3.3

*Consider the renewal risk process* (1.3) *with END risk sequence*. *In addition to Assumption* 3.1 *with* $\mathbf {E}T^{*} <\infty$, *suppose that* $F\in\mathcal{C}$, $\mathbf {E}T=1/\lambda<\infty$, $\mathbf {E}|Y|^{\beta}<\infty$ *for some* $\beta>\alpha>1$ *and* $\operatorname {\mathbf {Var}}[T]<\infty $. *If* (3.7) *holds*, *then*, *for any given* $\gamma>0$, (3.8) *holds uniformly for all* $x\geq\gamma b(t)$, *where* $b(t)=a(\lambda t)$ *is a positive function satisfying the conditions in Theorem *3.2.

Then we get the following result for precise large deviations.

#### Corollary 3.2

*Consider the renewal risk process* (1.3) *with END risk sequence*. *In addition to Assumption* 3.1 *with* $\mathbf {E}T^{*} <\infty$, *suppose that* $F\in\mathcal{C}$, $\mathbf {E}T=1/\lambda<\infty$, $\mathbf {E}|Y|^{\beta}<\infty$ *for some* $\beta>1$ *and* $\operatorname {\mathbf {Var}}[T]<\infty$. *then*, *for any given* $\gamma>0$, (3.8) *holds uniformly for all* $x\geq\gamma t$.

The proof of Theorem 3.3 is similar to that of Theorem 3.2.

#### Remark 3.3

For the END claims $\{Y_{n}, n\geq1\}$, in addition to Assumption 3.1, one needs additional conditions to ensure the sequence $\{T_{n}, n\geq1\}$ to be *k*-dependent.

## Conclusions

Motivated by Ma et al. [22], this paper investigates a nonstandard renewal counting process with k-dependent inter-arrival times, and obtains some important limit properties. We obtained the tail of the exponential moment of the counting process, which is crucial in many situations. We considered the applications of the main results in risk theory, and derived the formulas of precise large deviations and moderate deviations of the web renewal risk process. These results allow applications in various natural and social sciences.

Many topics based on the web renewal risk process shall be investigated. For example, Li et al. [16] studied a stochastic interest model based on compound Poisson process, and it is of interest to study the problem based on the web renewal risk process.

## Methods/experimental

Not applicable.

## Appendix 1

First we will give some lemmas needed for proving Theorem 3.2.

### Lemma A.1

*Let* $\{Y_{n}, n>0\}$ *be an END sequence with a common distribution function* $F\in\mathcal{C}$ *and finite mean* *μ*. *If there exists some* $\beta>\alpha>1$ *such that* $\mathbf {E}|Y_{1}|^{\beta }<\infty$, *then*, *for any* $\gamma> 0$, *we have uniformly for* $x\geq\gamma a(n)$

$$\Pr(S_{n}-n\mu>x)\sim n\overline{F}(x), \quad\textit{as } n\to\infty, $$

*where* $S_{n}$ *is the partial sum of* $\{Y_{k},k>0\}$.

Lemma A.1 is the moderate deviations for partial sums of END random variables, and one can see Theorem 2.1 of Liu [19] for more details.

### Lemma A.2

*Let* $\{Y_{n}, n\geq1\}$ *be an END sequence with common d*.*f*. *F* *and upper Matuszewska index* $J^{+}_{F}<\infty$, *and* $T_{n}$ *be dependent on* $Y_{n-1}$ *and* $Y_{n}$ *and independent of* $Y_{1}, Y_{2}, \dots, Y_{n-2}, Y_{n+1},\dots$. *Then*, *for every* $p > J^{+}_{F}$, *there is some constant* $C > 0$ *such that*, *uniformly for all* $x\geq0, t \geq0$ *and* $n\geq2$,

A.1 $$ \Pr \Biggl(\sum_{k=1}^{n}Y_{k}>x, \tau_{n}\leq t \Biggr)\leq C n^{p+1}\overline{F}(x)\Pr( \tau_{n-2}\leq t). $$

### Proof

From the nonnegativity of *T* and the independence between $T_{n}$ and $Y_{1}, \dots, Y_{n-2}$, $Y_{n+1},\dots$, we can get

A.2 $$\begin{aligned} \Pr \Biggl(\sum_{k=1}^{n}Y_{k}>x, \tau_{n}\leq t \Biggr) &\leq\sum _{k=1}^{n}\Pr \Biggl(Y_{k}> \frac{x}{n}, \sum_{i=1, i\neq k,i\neq k+1}^{n}T_{i} \leq t \Biggr) \\ &= n \Pr \biggl(Y>\frac{x}{n} \biggr)\Pr(\tau_{n-2}\leq t). \end{aligned}$$

Inequality (3.1) implies that, for any fixed $p > J^{+}_{F}$, there are some large positive constants $C_{1}$ and $x_{0}$ such that the inequality $\Pr(Y > x/n) \leq C_{1}n^{p}\overline {F}(x)$ holds for all $x\geq nx_{0}$. This, together with (3.2), gives

A.3 $$ \Pr \biggl(Y>\frac{x}{n} \biggr)\leq\frac{(n x_{0})^{p}}{x^{p}}+C_{1}n^{p} \overline{F}(x)\leq Cn^{p}\overline{F}(x),\quad C>C_{1}. $$

Substituting (A.3) into (A.2) yields the desired inequality (A.1). □

Based on Assumption 3.2, we construct a special generalized double-delayed renewal counting process. Set

$$\tau_{n}^{*}=T_{1}^{*}+T_{2}^{*}+\sum _{k=3}^{n}T_{k},\quad n>1, $$

where the nonnegative random variables $T_{1}^{*}$ and $T_{2}^{*}$, which are stochastically bounded by the above-mentioned $r.v$. $T^{*}$, are independent of all sources of randomness and have the same distributions as *T*. We note that $T_{1}^{*}$ and $T_{2}^{*}$ are not necessarily independent. Define the counting process

A.4 $$ N_{t}^{*}=\sup\bigl\{ n: \tau^{*}_{n}\leq t\bigr\} ,\quad t\geq0. $$

The following lemma establishes the law of large numbers for $\{ N_{t}^{*},t\geq0\}$.

### Lemma A.3

*We assume that* $T_{n}$, $n\geq 1$ *are* 1-*dependent and identically distributed with mean* $\lambda<\infty$ *and finite variation*. *We suppose that* $T_{n}$ *is stochastically bounded by the random variable* $T^{*}$, $\operatorname {\mathbf {Var}}[T^{*}]\in (0, \infty)$. *If* (3.7) *holds*, *then*, *for every function* $c(t):[0,\infty)\rightarrow[0,\infty )$ *with* $c(t)\rightarrow\infty$ *as* $t\rightarrow\infty$, *the result*

$$\frac{N_{t}^{*}-\lambda t}{b(t)}\stackrel{P}{\longrightarrow} 0, \quad t\rightarrow\infty, $$

*holds uniformly for all* $x\geq c(t)$, *i*.*e*., *for any* $0<\epsilon <\lambda$,

A.5 $$ \lim_{t\rightarrow\infty}\sup_{x\geq c(t)}\Pr \biggl( \biggl\vert \frac {N_{t}^{*}-\lambda t}{b(t)} \biggr\vert >\epsilon \biggr) =0. $$

### Proof

Observe that, for all sufficiently large *t*,

$$\begin{aligned} & \Pr \biggl( \biggl\vert \frac{N_{t}^{*}-\lambda t}{b(t)} \biggr\vert >\epsilon \biggr) \\ &\quad=\Pr(N_{t}^{*}< \lambda t-\epsilon b(t)+\Pr(N_{t}^{*}\geq \lambda t+\epsilon b(t) \\ &\quad\leq \Pr\Biggl(2T^{*}+ \sum _{k=3}^{\lfloor\lambda t-\epsilon b(t)\rfloor}T_{k}>t\Biggr)+ \Pr\Biggl(\sum _{k=3}^{\lfloor\lambda t+\epsilon b(t)\rfloor}T_{k}\leq t\Biggr). \end{aligned}$$

By the Markov law of large numbers for the partial sums $\sum_{k=1}^{n}T_{k}$, both probabilities on the right-hand side above converge to zero as $t\rightarrow\infty$. Thus, Eq. (A.5) holds. □

Now, it is ready to prove Theorem 3.2. We first prove

A.6 $$ \Pr(S_{t}-\mu\lambda t>x)\gtrsim\lambda t \overline{F}(x) $$

and

A.7 $$ \Pr(S_{t}-\mu\lambda t>x)\lesssim\lambda t \overline{F}(x) $$

separately. The proofs of these two relations will complete the proof of Theorem 3.2.

Throughout this section, unless otherwise stated, every limit relation is understood as valid uniformly for all $x\geq\gamma b(t)$ as $t\rightarrow\infty$.

### 1.1 Proof of (A.6)

Fixing $\epsilon\in(0,1)$ and $\nu>1$, we can derive

$$\begin{aligned} & \Pr(S_{t}-\mu\lambda t>x) \\ &\quad\geq\sum_{n=\lambda t-\epsilon b(t)}^{\lambda t+\epsilon b(t)}\Pr \Biggl(S_{n}-\mu\lambda t>x, N_{t}=n, \bigvee _{i=1}^{n}Y_{k}> \nu x \Biggr) \\ &\quad\geq\sum_{n=\lambda t-\epsilon b(t)}^{\lambda t+\epsilon b(t)}\sum _{i=1}^{n}\Pr \bigl(S_{n,i}-\mu\lambda t>(1- \nu)x, N_{t}=n, Y_{i}>\nu x \bigr) \\ &\qquad{} -\sum_{n=\lambda t-\epsilon b(t)}^{\lambda t+\epsilon b(t)}\sum _{1\leq i< j\leq n}\Pr ( N_{t}=n, Y_{i}> \nu x, Y_{j}> \nu x ) \\ & \quad=: I_{1}(x,t)-I_{2}(x,t), \end{aligned}$$

where $S_{n,i}=S_{n}-Y_{i}$.

We deal with $I_{1}$ and $I_{2}$ separately. Firstly, according to Liu [19], for some positive *ϵ* small enough so that $(1-\nu)\gamma+\epsilon\mu<0$, $0<\rho<1$,

$$\Pr \bigl(S_{n,i}-\mu\lambda t\leq(1-\nu)x, Y_{i}>\nu x \bigr)\leq o\bigl(\overline{F}(x)\bigr) $$

holds uniformly for all $x\geq\gamma a(n)$ and large *n*. Then we have

$$\begin{aligned} I_{1}(x,t) ={}&\sum_{n=\lambda t-\epsilon b(t)}^{\lambda t+\epsilon b(t)}\sum _{i=1}^{n} \Pr \bigl(S_{n,i}-\mu \lambda t>(1-\nu)x, N_{t}=n| Y_{i}>\nu x \bigr)\Pr (Y_{i}>\nu x) \\ \geq{}&\sum_{n=\lambda t-\epsilon b(t)}^{\lambda t+\epsilon b(t)}\sum _{i=1}^{n} \bigl(\Pr\bigl(S_{n,i}-\mu\lambda t>(1-\nu)x| Y_{i}>\nu x\bigr)\\ &{}-\Pr(N_{t}\neq n| Y_{i}>\nu x) \bigr) \Pr(Y_{i}>\nu x) \\ \geq{}&\bigl(\lambda t-\epsilon b(t)\bigr) \biggl(\overline{F}(\nu x)-o\bigl( \overline {F}(x)\bigr) - \Pr \biggl( \biggl\vert \frac{N_{t}^{*}-\lambda t}{b(t)} \biggr\vert >\epsilon \biggr) \overline{F}(\nu x) \biggr), \end{aligned}$$

where $N_{t}^{*}$ is a generalized double-delayed renewal counting process constructed as in (A.4). Hence Lemma A.3 and $F\in \mathcal{C}$ imply

A.8 $$\begin{aligned} &\liminf_{t\rightarrow\infty}\inf _{x\geq\gamma b(t)}\frac {I_{1}(x,t)}{\lambda t\overline{F}(x)} \\ &\quad\geq\lim_{\varepsilon\downarrow0}\lim_{\nu\downarrow1}\liminf _{t\rightarrow\infty}\inf_{x\geq\gamma b(t)} \frac{\lambda t-\epsilon b(t)}{\lambda t} \frac{\overline{F}(\nu x)-o(\overline{F}(x)) - \Pr ( \vert \frac{N_{t}^{*}-\lambda t}{b(t)} \vert >\epsilon ) \overline{F}(\nu x) }{\overline {F}(x)} \\ &\quad =1. \end{aligned}$$

As for $I_{2}(x,t)$, by virtue of the END property and Remark 3.2, it is easy to get

A.9 $$ \limsup_{t\rightarrow\infty}\sup_{x\geq\gamma b(t)} \frac {I_{2}}{\lambda t\overline{F}(x)}=0. $$

Equation (A.9), together with (A.8), ensures that (A.6) holds.

### 1.2 Proof of (A.7)

We now consider the case $\Pr(S_{t}-\mu\lambda t>x)\lesssim\lambda t \overline{F}(x)$. We can rewrite

A.10 $$ \Pr(S_{t}-\mu\lambda t>x)=J_{1}+J_{2} $$

using

$$\begin{aligned} &J_{1}(x,t)=\Pr \Biggl(\sum^{N_{t}}_{k=1}Y_{k}- \mu\lambda t>x, N_{t} \leq \lambda t+\epsilon b(t) \Biggr), \\ &J_{2}(x,t)= \Pr \Biggl( \sum^{N_{t}}_{k=1}Y_{k}- \mu\lambda t>x, N_{t} > \lambda t+\epsilon b(t) \Biggr). \end{aligned}$$

By Lemma A.1, we get

A.11 $$ J_{1}(x,t)\leq\Pr \biggl(\sum _{1\leq k\leq\lambda t+\epsilon b(t)}Y_{k}-\mu\lambda t>x \biggr) \leq\bigl(\lambda t+\epsilon b(t)\bigr) \overline{F}\bigl(x(1-\epsilon/\gamma ) \bigr). $$

By Lemma A.2 and Theorem 2.2, we have

A.12 $$ J_{2}(x,t)= \sum _{ n>(1+\varepsilon)\lambda t}\Pr (S_{n}-\mu \lambda t>x, N_{t}=n )\leq o\bigl(\lambda t\overline{F}(x)\bigr). $$

Substituting (A.11) and (A.12) into (A.10) yields

$$ \Pr(S_{t}-\mu\lambda t>x) \lesssim\bigl(\lambda t+\epsilon b(t)\bigr) \overline {F}\bigl(x(1-\epsilon/\gamma)\bigr)+o\bigl(\lambda t\overline{F}(x) \bigr). $$

By the arbitrariness of *ϵ* and the condition $F\in\mathcal {C}$, we obtain (A.7).

The proofs of (A.6) and (A.7) complete the proof of Theorem 3.2.
